# The Feasibility and Efficacy of Social Cognition and Interaction Training for Outpatients With Schizophrenia in Japan: A Multicenter Randomized Clinical Trial

**DOI:** 10.3389/fpsyt.2019.00589

**Published:** 2019-08-23

**Authors:** Ayako Kanie, Akiko Kikuchi, Daisuke Haga, Yuki Tanaka, Akina Ishida, Yuko Yorozuya, Yasuhiro Matsuda, Tsubasa Morimoto, Tomoharu Fukuoka, Satoru Takazawa, Kumiko Hagiya, Sachiyo Ozawa, Kazuhiko Iwata, Emi Ikebuchi, Takahiro Nemoto, David L. Roberts, Kazuyuki Nakagome

**Affiliations:** ^1^Department of Cognitive Behavior Therapy, National Center of Neurology and Psychiatry, Kodaira, Japan; ^2^Department of Community Mental Health & Law, National Institute of Mental Health, National Center of Neurology and Psychiatry, Kodaira, Japan; ^3^One More Toyonaka, Toyonaka, Japan; ^4^Department of Neuropsychiatry, Toho University School of Medicine, Ota-ku, Japan; ^5^Department of Psychiatry, Nara Medical University School of Medicine, Kashihara, Japan; ^6^Department of Psychiatry, Inuyama Hospital, Inuyama, Japan; ^7^National Center of Neurology and Psychiatry Hospital, Kodaira, Japan; ^8^Osaka Psychiatric Medical Center, Hirakata, Japan; ^9^Department of Psychiatry, Teikyo University School of Medicine, Itabashi-ku, Japan; ^10^Department of Psychiatry, University of Texas Health Science Center at San Antonio, San Antonio, TX, United States

**Keywords:** social cognition and interaction training, social cognition, schizophrenia, theory of mind, randomized clinical trial

## Abstract

**Background:** Schizophrenia is a disabling illness. Social cognition and interaction training (SCIT) seeks to improve patients’ social functioning by alleviating deficits in social cognition. SCIT has shown promise in improving social cognition in patients with schizophrenia, but has not yet been studied in Japan.

**Design:** An assessor-masked, randomized, parallel-group clinical trial was conducted to compare the feasibility and efficacy of SCIT with treatment as usual (TAU).

**Setting:** Participants were recruited from outpatient clinics at the National Center of Neurology and Psychiatry and four other hospitals in Japan.

**Participants:** Seventy-two patients diagnosed with schizophrenia or schizoaffective disorder consented to participate in the trial.

**Procedure:** Participants were randomly allocated to either a SCIT subgroup or a TAU subgroup. SCIT is a manual-based group intervention that is delivered in 20–24-h-long weekly sessions. Groups include two to three clinicians and four to eight patients.

**Hypotheses:** We hypothesized that SCIT would be found to be feasible and that patients who were randomized to receive SCIT would exhibit improvements in social cognition.

**Results:** Data from 32 participants in each subgroup were entered into analyses. The persistence rate in the SCIT subgroup was 88.9%, and the average attendance rate was 87.0%. Intrinsic motivation was significantly higher in the SCIT subgroup than the TAU group during the first half of the program. Mixed effects modeling of various outcome measures revealed no significant interaction between measurement timepoint and group in any measures, including social cognition, neurocognition, symptom severity, and social functioning. In the case of the social cognition measure, significant change was observed only in the SCIT subgroup; however, the interaction between timepoint and group failed to reach significance. In an exploratory subgroup analysis, a shorter duration of illness was found to be associated with significantly better improvement on the social cognition measure in the SCIT subgroup compared with the TAU subgroup.

**Conclusions:** In terms of the primary objective, the relatively low dropout rate observed in the present study suggests that SCIT is feasible and well tolerated by patients with schizophrenia in Japan. This view is also supported by participants’ relatively high attendance and intrinsic motivation.

## Introduction

Schizophrenia is a chronic, severe, and disabling illness that affects approximately 1% of individuals in the population, with onset usually during late adolescence or early adulthood (1). It is characterized by a combination of positive, negative, and affective symptoms, but cognitive deficits, including impairment of neuro- and social cognitions, are the core symptoms that affect patients’ everyday lives. It has been pointed out that the variance in patients’ social and community functional outcomes can be explained by composite measures of neurocognition (2). Pharmacological interventions have shown some ability to improve neurocognition, but their capacity to improve patients’ social and community functional outcomes is extremely limited (3).

It has been reported that at most 20% to 40% of the variance in functional outcomes can be explained by composite measures of neurocognition (4). More recently, there is a growing body of evidence that social cognition may serve as a mediator between neurocognition and functional outcome (5–9). *Social cognition* refers to the cognitive and emotional functions required to understand and predict other people’s mental states and behaviors (10). Patients with schizophrenia experience substantial deficits in social cognition across multiple domains. The most commonly studied domains include emotion perception, social perception, attributional bias, jumping to conclusions, metacognition, and theory of mind (ToM) (11). Therefore, improving social cognition may well lead to improvement in social functioning.

Social cognition and interaction training (SCIT), which was developed by Penn and colleagues, is a program for social cognitive rehabilitation in schizophrenia (12). Compared to other psychosocial interventions that have been designed to target specific social cognitive impairments to the exclusion of other domains (13, 14), SCIT targets a broader range of social cognition. SCIT includes training sessions focusing on emotion perception, distinguishing facts from guesses, generating alternative interpretations of social situations, and avoiding jumping to conclusions, which are designed to increase flexibility in attributional style and improve metacognition. SCIT is theorized to improve social cognition through a combination of rehearsal-based “bottom-up” learning and acquisition of social cognitive strategies that are consciously deployed by patients in social situations.

SCIT has shown promise in improving social cognition in patients with schizophrenia in various regions of the world. However, this research has consisted largely of studies with small samples, and no study has yet been carried out in Japan. Across a series of small trials conducted by its developers, SCIT has shown evidence of feasibility and tolerability in community settings (15), efficacy in improving social cognition and social functioning (16, 17), and some evidence that treatment gains persist over a 6-month follow-up period (18). SCIT has also yielded promising findings when implemented by independent research groups in Australia, Hong Kong, mainland China, Turkey, Spain, Israel, and Finland (19–25).

The main aims of the present study were to examine the feasibility of SCIT, whether patients would exhibit intrinsic motivation for the program, its efficacy in improving social cognition deficits, and possible predictors of improvements in social cognition in Japanese patients in a real-world outpatient setting. Specifically, we hypothesized that the social cognition measures, including facial emotion identification, theory of mind, attributional style, including hostile bias, and metacognition, would be improved since these domains are targeted in SCIT. We also explored the effects of SCIT on social functioning, positive and negative symptoms, and neurocognition. As already noted, since impairment in social cognition has been shown to have a significant impact on functional outcome in patients with schizophrenia, (4) we expected that social functioning may be improved along with improvement in social cognition. Moreover, theoretically, patients with enhanced social cognitive skills may show better coping strategies and be better able to gain social support to decrease their level of stress, and consequently, may be less likely to show positive and negative symptoms. Finally, as SCIT requires the patients to focus attention on facial expression and the social interaction vignettes, and also to remember what they learned in each session, treatment effects may well generalize from social cognitive to neurocognitive domains. However, we would expect this effect to be small because the program was not any more specific to neurocognition than other treatment programs. Although improvements in social functioning, positive and negative symptoms and neurocognition would be consistent with the SCIT intervention approach; these are not the primary treatment targets of SCIT, and so, we did not formally hypothesize change in these domains in this initial trial.

## Materials and Methods

### Participants

Participants were 73 patients treated between August 2013 and July 2017. Sample characteristics are presented in **Table 1**. Inclusion in the trial required that the patient be at least 20 years old and no more than 65 years old, and that they meet the diagnostic criteria for schizophrenia or schizoaffective disorder specified in the Diagnostic and Statistical Manual of Mental Disorders, fourth edition, text revised (26). Participants were recruited from outpatient clinics at the National Center of Neurology and Psychiatry (NCNP), Sawa Hospital, Toho University Hospital, Inuyama Hospital, and Nara Medical University Hospital in Japan. All patients were attending psychosocial treatment at least once a week at the time of the study and had the capacity to give written informed consent.

**Table 1:** Participants’ sociodemographic and clinical profiles. Continuous variables are presented in the form *M* ± *SD*.

Variables	SCIT subgroup (*n* = 32)	TAU subgroup (*n* = 32)
Sex (*n* male)	20	17
Age (years)	35.5 ± 10.1.5	37.5 ± 9.6
Duration of illness (months)	174.7 ± 118.4	161.4 ± 124.2
Daily dosage level (CPZ equiv.)	705.9 ± 506.3	559.0 ± 450.4
Years of education	13.1 ± 1.7	13.9 ± 2.1
Daily activities (*n*)		
Competitive work	17	14
Non-competitive work	4	5
Homemaker	1	2
Student	2	2
Community workshop	1	1
Day care center	2	2
Social withdrawal	5	2
Other	0	4
PANSS score		
Total	64.8 ± 18.2	63.8 ± 19.0
Positive	15.4 ± 5.1	15.4 ± 5.9
Negative	16.2 ± 5.4	16.7 ± 5.9
General	33.2 ± 9.8	31.7 ± 9.7
GAF score	51.6 ± 8.8	53.4 ± 9.5
JART score	100.5 ± 10.9	102.3 ± 12.2
IMI score		
BL–3M	104.7 ± 21.6**	89.3 ± 23.1
3M–6M	100.1 ± 24.3	91.2 ± 20.0
Attendance rate (%)		
BL–3M	87.4 ± 15.9	N/A
3M–6M	86.4 ± 18.7	N/A
BL–6M	87.0 ± 14.8	N/A

**P < 0.01 in student’s t–test, SCIT subgroup vs. TAU subgroup, P < 0.01.PANSS, Positive and Negative Syndrome Scale; GAF, Global Assessment of Function; JART, Japanese Adult Reading Test; IMI, Intrinsic Motivation Inventory; BL, baseline; 3M, 3-month timepoint; 6M, 6-month timepoint; SCIT, Social Cognition and Interaction Training; TAU, treatment as usual.

Patients were excluded from participation in the study if they presented at screening with serious suicidal ideation or life-threatening, severe, or unstable physical disorders; had a history of substance or alcohol use disorder within 3 months prior to screening; or had an estimated IQ lower than 70, as assessed by the Japanese Adult Reading Test (JART) (27). Patients for whom participation in the study was deemed to be inappropriate by the doctor in charge and/or the investigator for any other reason were also excluded.

### Study Setting

The SCIT-J study was conducted at multiple sites: the NCNP, a general hospital specializing in psychiatry and neurology located in the suburbs of Tokyo; Sawa Hospital, a psychiatry hospital located in Osaka; Toho University School of Medicine, a university hospital located in Tokyo; Inuyama Hospital, a psychiatry hospital located in Aichi; and Nara Medical University, a university hospital located in Nara.

### Procedure

Seventy-three potential participants were referred to the SCIT-J trial. Seventy-two patients consented to participate in the trial and met the enrolment criteria. Participants were randomly allocated to either a SCIT subgroup or a treatment as usual (TAU) subgroup in a 1:1 ratio using a computer-generated sequence obtained online. Restricted and adapted randomization (i.e., minimization) was used to minimize imbalances in age (30 or older *vs*. under 30). Throughout the whole process, no rater was able to access information that could possibly reveal a participant’s subgroup allocation.

In order to adapt the SCIT program to the Japanese culture and people, the SCIT manual was translated into Japanese with the help of one of the authors (DR), who is one of the developers of SCIT (28). Additionally, social interaction scenarios used in the program were recreated using Japanese actors, following the original scripts. Although we did not follow the translation-back translation methodology, we received a training and were given supervision from the developers of SCIT and also had multiple sessions of interactive discussion in adapting the program to Japanese culture.

SCIT is a manual-based group intervention that is delivered in 20–24 h-long weekly sessions. Groups consist of two to three clinicians (psychiatrists, clinical psychologists, and/or occupational therapists) and four to eight patients. In the present study, treatment sessions were conducted by clinicians (psychiatrists, clinical psychologists, and/or occupational therapists) who were all trained extensively in a two-day hands-on training session and received ongoing supervision. Treatment fidelity was monitored using the Therapist Adherence Rating Scale for SCIT (28). SCIT is made up of three modules, targeting 1) emotion perception training, which consists of defining emotions, emotion mimicry training, and understanding paranoia (seven sessions); 2) figuring out situations, which consists of distinguishing facts from guesses and avoiding jumping to conclusions (eight sessions); and 3) integration (five to nine sessions).

During the study period, the TAU subgroup attended other psychosocial treatment programs other than SCIT and cognitive remediation therapy at least once a week. For all participants, the use of psychotropic medication was not necessarily fixed; rather, it was flexible according to their clinical state. Participants’ antipsychotic medications at baseline are presented in **Table 2**; mean daily dosage levels (CPZ equiv.) did not differ significantly between the two subgroups. We compensated each participant for their time with a payment of 5,000 JPY (approximately 38.8 EUR) at each assessment.

**Table 2:** Number of patients prescribed each antipsychotic medication at baseline.

Antipsychotic drug	SCIT subgroup (*n* = 32)	TAU subgroup (*n* = 32)
Olanzapine	11	10
Aripiprazole	10	12
Risperidone	7	8
Blonanserin	5	1
Paliperidone	4	4
Levomepromazine	4	2
Quetiapine	3	2
Risperidone LAI	3	0
Zotepine	2	3
Perospirone	2	1
Haloperidol	1	0
Chlorpromazine	1	2
Bromperidol	0	1
Sultopride	0	1
Mean daily dosage (CPZ equiv.)	705.9 ± 506.3	559.0 ± 450.4

### Trial Design

The study consisted of an assessor-masked, randomized, parallel-group clinical trial that compared the feasibility and efficacy of SCIT in conjunction with TAU to TAU alone. Assessment measures were administered to all patients at baseline (between 4 and 1 weeks prior to the start of the SCIT treatment program), at a 3-month interim assessment (after 12 ± 2 weeks), and at a 6-month endpoint assessment (after 24 ± 2 weeks; **Table 3**).

**Table 3:** Trial design, showing when each measure was administered.

Measure	Screening	Baseline	3-month interim timepoint	6-month endpoint
Sociodemographic and clinical profile	•			
Diagnosis (DSM-IV-TR)	•			
Japanese Adult Reading Test	•			
Positive and Negative Syndrome Scale		•	•	•
Brief Assessment of Cognition Schizophrenia		•	•	•
Face Emotion Selection Test		•	•	•
Hinting Task		•	•	•
Social Cognition Screening Questionnaire		•	•	•
Social Functioning Scale		•	•	•
Global Assessment of Functioning		•	•	•
Intrinsic Motivation Inventory			•	•

Ethical approval was obtained for the study (Ethical Committee of the National Centre of Neurology and Psychiatry), and the trial was registered with the University Hospital Medical Information Network (UMIN000011240; URL: https://upload.umin.ac.jp/cgi-open-bin/ctr/ctr_view.cgi?recptno=R000013171)

### Measures

#### Feasibility

One of the primary outcome measures was feasibility, which was estimated by a) persistence rate at the 6-month endpoint, b) treatment adherence rate (attendance rate), and c) intrinsic motivation for SCIT (at the interim timepoint and endpoint), as assessed by the intrinsic motivation inventory (IMI) (29). The IMI (29) is a 21-item self-report measure assessing participants’ subjective experiences in relation to a target program that they are engaging with. Subscales are interest/enjoyment, choice, and value/usefulness (range: 21–144, higher scores indicate stronger intrinsic motivation level).

#### Social Cognition

##### The Social Cognition Screening Questionnaire

Another primary outcome measure was change in social cognition, as assessed by total scores on the Social Cognition Screening Questionnaire (SCSQ) (30). The SCSQ is a measure consisting of five subscales assessing verbal memory, schematic inference, ToM, metacognition, and hostility bias. The task comprises 10 short vignettes presenting an interaction between a fictional character and the study participant. Each vignette was read aloud by the tester. The tester then asked the participant to answer “yes” or “no” to three questions about the vignette, addressing verbal memory, schematic inference, and ToM. ToM items were designed to assess both ToM and hostile attributional bias. Scores for the verbal memory, schematic inference, and ToM subscales were computed by counting the number of correct answers to corresponding items (range: 0–10; higher scores indicate better performance). Scores on the hostility bias subscale were calculated by counting the number of instances in which the participant erroneously inferred that the character in the vignette had negative thoughts or feelings towards the participant (range: 0–5; higher scores indicate stronger bias). With regard to metacognition scores, if the subject answered correctly on the corresponding “yes” or “no” question, a score of 1 was given. If the subject answered incorrectly on the question, a score of 0 was given if he/she answered that he/she was “very sure,” 0.33 for “pretty sure,” 0.66 for “a little unsure,” and 1 for “not sure at all.” The total metacognition score was obtained by summing the scores for the 10 vignettes (range 0–10; higher scores indicate better metacognitive ability). The total SCSQ score was calculated by summing all subscale scores, except the hostility bias subscale, because the items used for calculating this score overlapped with those used to calculate ToM score (range 0–40; higher scores indicate better social cognition). We used the Japanese version of SCSQ, which was translated and adapted to Japanese language and culture and validated in our previous study (31).

The SCSQ addresses multiple subdomains of social cognition including theory of mind, hostile attributional bias, and metacognition within a reasonable time period of about 15 min. In our previous validation study (31), it was demonstrated that the SCSQ showed more robust discrimination between patients with schizophrenia and healthy controls than other measures of social cognition. Moreover, the SCSQ subscales showed both fair convergent and discriminant validity as indicated by its significant relationship with measures which are thought to address the same subdomains of social cognition and not with those that are thought to measure other domains. Finally, the SCSQ theory of mind subscale scores in particular was significantly correlated with the scores of four domains of the Social Functioning Scale (SFS) (32), which supports the ecological validity of the SCSQ. Based on these findings, we decided to use the SCSQ as a primary measure but also to use the conventional Hinting task (33) as a secondary measure.

##### The Face Emotion Selection Test

The Face Emotion Selection Test (FEST) (34) is a measure used to assess emotion perception, consisting of 21 photographs of human faces depicting seven different emotions (happiness, sadness, anger, disgust, surprise, fear, and a neutral expression), with three images for each emotion. The photographs of Japanese actors were presented in a random order to the participant, and in each case, the participant selected one of the seven emotions; these were listed below the stimulus photograph and printed in Japanese. The score for each emotion was the number of correct responses (range: 0–3) to faces depicting that emotion. The total score was the sum of all scores for individual emotions (range: 0–21, higher scores indicate better face emotion perception).

In our previous study (34), it was demonstrated that the patients with schizophrenia performed significantly worse on the FEST compared to healthy control subjects. The FEST total score was significantly positively correlated with scores of the Brief Assessment of Cognition in Schizophrenia (35) attention subscale, Hinting Task, SCSQ verbal memory, and metacognition subscales.

##### The Hinting Task

The Hinting Task (33) is a measure assessing ToM and requires participants to infer the real intentions behind indirect speech. The task comprised 10 short passages presenting an interaction between two characters in which dialogue produced by one of the characters provided a hint at an underlying message. The participant was then asked what the character really meant when s/he uttered the hint. If the participant failed to give the correct response, an additional hint was given, and the question was asked again. Therefore, a correct response scored either 2 or 1, depending on whether the extra hint was given. The total score was obtained by summing the participant’s scores for each passage (range: 0–20, higher scores indicate better theory of mind capacity).

We used the Japanese version of the Hinting task, which was translated and adapted to Japanese language and culture, and its psychometric properties were verified by showing a significant relationship with the SCSQ ToM subscale and also a significant difference between the patients with schizophrenia and healthy controls in our previous study (31).

#### The Brief Assessment of Cognition in Schizophrenia

The BACS (35) is a measure assessing neurocognitive function in six cognitive domains: verbal memory, working memory, motor speed, verbal fluency, attention, and executive function. We used the Japanese version of the BACS (36). The primary BACS measure, namely, the total score, was standardized by computing *z*-scores with reference to a distribution in which the mean score of Japanese healthy controls was set at 0 and the standard deviation set at 1.

In a validation study of the Japanese version of the BACS, internal consistency was acceptable with Cronbach’s alpha of 0.77 and showed a good construct validity with the composite score being significantly correlated with standard neurocognitive tests in general (36). Accordingly, the Japanese version of the BACS is widely accepted in Japan as a practical scale to evaluate cognitive function.

#### The Social Functioning Scale

The SFS (32) is a measure assessing social functioning across seven domains: social engagement, interpersonal communication, independence-performance, recreation, social activities, independence-competence, and occupation (with higher scores indicating a higher level of functioning); each domain is assessed either by the participant themselves or by an informant. In the present study, we used self-report rather than informant interview. The score for each domain was computed by summing the item scores in the corresponding domain; the total score was the sum of all seven domain scores (range: 0–222, higher scores indicate better social functioning level). We used the Japanese version of SFS, which was validated in a previous study (37).

In addition to the above measures, the PANSS (38) was used to assess symptom severity and the GAF to assess functioning (39).

### Statistical Analysis

We used linear mixed effects modeling (conducted using SAS 9.4; SAS Institute Inc., Cary, NC, USA) to compare the SCIT and TAU subgroups over time on outcome variables reflecting their social cognition, neurocognitive functioning, symptom levels, and social functioning. The model included fixed main effects of treatment subgroup (TAU *vs*. SCIT) and timepoint (baseline *vs*. 3-month interim timepoint *vs*. 6-month endpoint), and a treatment subgroup × timepoint interaction. In addition, random effects for participants were included. A significant interaction term in such a model would imply a difference in the effect of the intervention between timepoints. It was hypothesized that the SCIT subgroup would show significant improvements over time on the SCSQ subscales relative to the TAU subgroup. Effect sizes are reported to supplement statistical tests, calculated using the following formula.

$$\frac{\left({\mathrm{SCIT}}_{6\text{ months}}-{\mathrm{SCIT}}_{\text{baseline}}\right)-\left({\mathrm{TAU}}_{6\text{ months}}-{\mathrm{TAU}}_{\text{baseline}}\right)}{\text{pooled SD of baseline scores}}$$

Next, in order to explore predictors of the effect of SCIT on SCSQ total scores, a stepwise multiple regression analysis was conducted. Improvement in total SCSQ score observed in the SCIT subgroup between the baseline, and 6-month endpoint assessments was entered into the analysis as a dependent variable, and sex, age, duration of illness, BACS score (baseline), SCSQ score (baseline), and IMI score (mean) were included as independent variables. When significant variables were identified in this analysis, the total sample was subdivided according to the median value of these variables, and a secondary analysis using linear mixed effects modeling was conducted for the subgroup that was likely to benefit from SCIT treatment.

The full analysis data set, including data from all participants who were assessed at least once after any kind of intervention, was submitted to data analyses.

## Results

### Sociodemographic and Clinical Profiles of Participants

**Figure 1:**
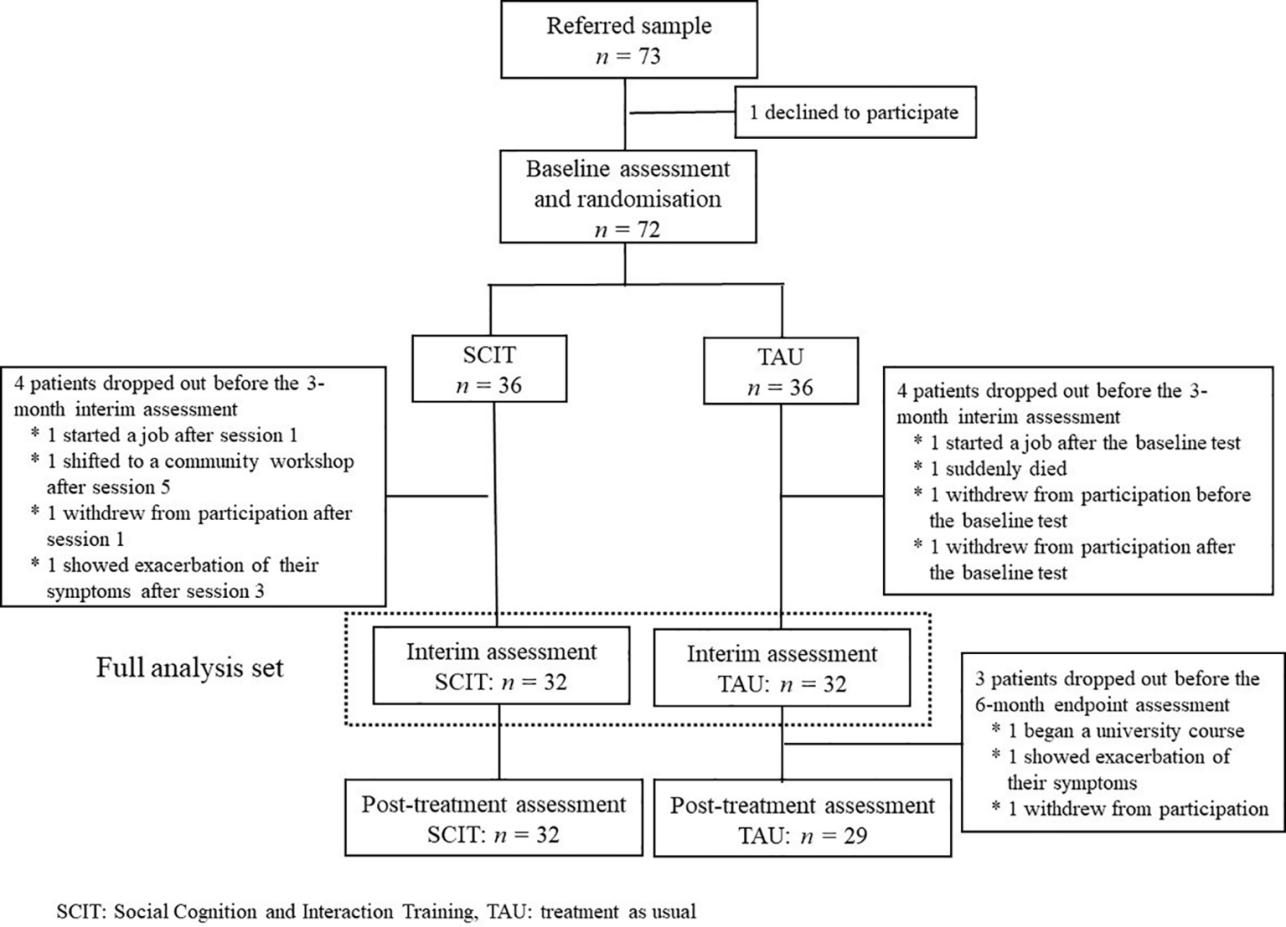
Consort flow diagram. Seventy-three patients were referred to the trial, and 72 agreed to participate. Thirty-six patients were allocated to the SCIT subgroup and 36 to the TAU subgroup. During the study, four patients in the SCIT subgroup and four patients in the TAU subgroup dropped out before the 3-month interim assessment. In addition, three in the TAU subgroup left the study before the 6-month endpoint assessment. Thus, data from 32 SCIT subgroup and 32 TAU subgroup participants were submitted to statistical analyses.

Seventy-three patients were referred as potential participants and 72 consented to participate in the trial. Thirty-six patients were allocated to the SCIT subgroup and 36 to the TAU subgroup. During the study, four patients in the SCIT subgroup dropped out before the 3-month interim assessment: one started a job after session 1, one shifted to a community workshop after session 5, one withdrew from participation after session 1, and one showed exacerbation of their symptoms after session 3. Four patients in the TAU subgroup also dropped out before the 3-month interim assessment: one started a job after the baseline test, one suddenly died, one withdrew from participation before the baseline test, and one withdrew from participation after the baseline test. In addition, three further patients in the TAU subgroup dropped out before the 6-month endpoint assessment: one began a university course, one showed exacerbation of their symptoms, and one withdrew from participation. Data from the eight patients who dropped out after randomization but before the 3-month interim assessment were omitted from the full analysis data set because they were not assessed after any intervention. Thus, the data from 32 SCIT subgroup and 32 TAU subgroup participants were submitted to statistical analyses (**Figure 1**).

The SCIT and the TAU subgroups did not differ significantly on any sociodemographic or clinical variables, such as sex, age, duration of illness, daily antipsychotic dosage level, years of education, daily activities, baseline level of symptoms, level of functioning, or IQ as estimated by the JART (**Table 1**). Average total PANSS scores showed that the patients were generally at a moderate level of symptom severity (40), and nearly half the patients were engaged in competitive work, suggesting that the participants in the present study were relatively high-functioning.

### Feasibility of SCIT

We estimated the feasibility of SCIT by using a) persistence rate at the 6-month endpoint, b) attendance rate, and c) IMI at the interim timepoint and endpoint.

The persistence rate in the SCIT subgroup was 88.9% (32/36), as only four out of 36 participants dropped out, all during the first half of the program, i.e., before the 3-month interim timepoint.

The average attendance rate at SCIT was 87.4% (*SD* = 15.9) between the baseline assessment and the 3-month interim assessment, 86.4% (*SD* = 18.7) between the 3-month interim assessment and the 6-month endpoint assessment, and 87.0% (*SD* = 14.8) over the entire period.

In addition, IMI scores for the first half of the program (between the baseline and 3-month interim timepoints) were significantly higher in the SCIT subgroup than in the TAU subgroup (*P* < 0.01). However, the groups did not differ significantly at end-point analysis.

### Change in the Total SCSQ Score

**Figure 2:**
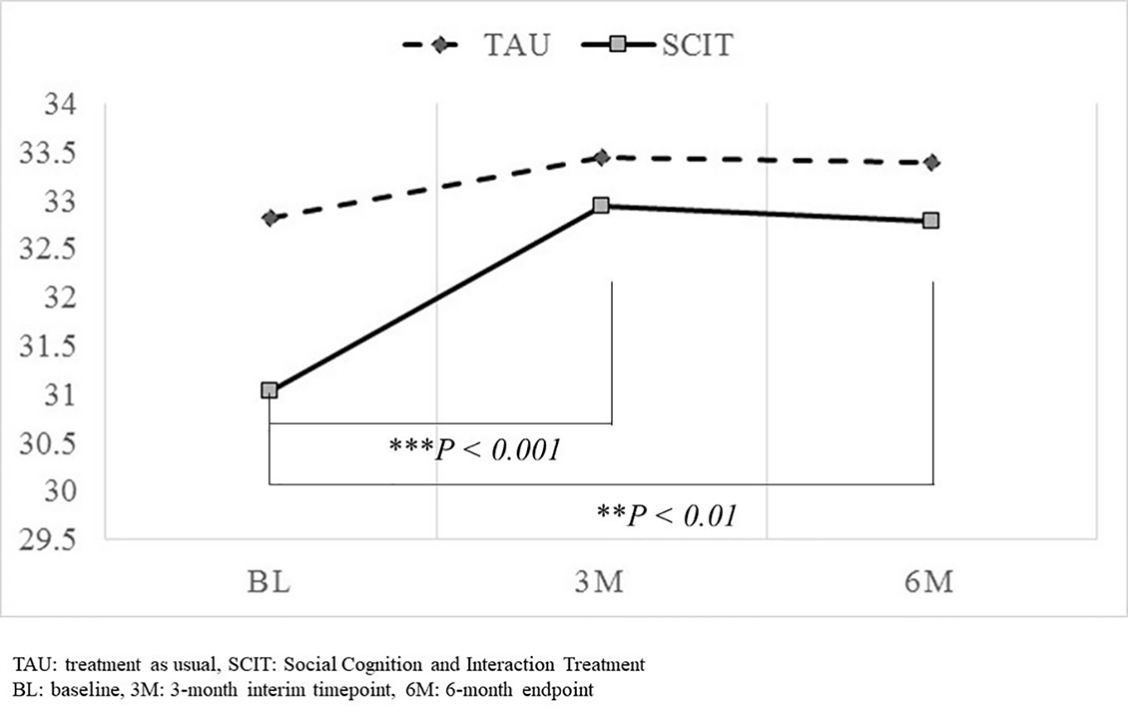
Comparison of changes in total SCSQ score from baseline to the 3-month interim timepoint and the 6-month endpoint. Significant change in total SCSQ scores was observed between the baseline and 3-month interim assessments, and also between the baseline and 6-month endpoint assessments only in the SCIT subgroup. However, the interaction between timepoint and group failed to reach significance. ** P<0.01, *** P<0.001.

The results of the mixed effects modeling revealed no significant interaction between timepoint and group in the total SCSQ score (**Table 4**). However, significant change was observed between the baseline and 3-month interim assessments, and also between the baseline and 6-month endpoint assessments, only in the SCIT subgroup (**Figure 2**).

**Table 4:** Results of mixed effects modeling of various outcomes.

Measure	Subgroup	BL	3M	6M	Main effect of time	Time × group interaction	Effect size (6M–BL)
SCSQ total	SCIT	31.04 (3.58)	32.94 (3.20)	32.79 (3.17)	*F*(2,123) = 6.94 *P* = 0.0014	*F*(2,123) = 1.74 *P* = 0.18	0.33
	TAU	32.83 (3.47)	33.45 (2.63)	33.40 (2.63)			
SCSQ ToM	SCIT	6.91 (1.20)	7.41 (1.54)	7.09 (1.38)	*F*(2,123) = 2.22 *P* = 0.11	*F*(2,123) = 0.32 *P* = 0.73	0.07
	TAU	7.22 (1.39)	7.47 (1.19)	7.31 (1.45)			
SCSQ HB	SCIT	1.66 (0.94)	1.28 (0.99)	1.08 (1.08)	*F*(2,123) = 1.79 *P* = 0.17	*F*(2,123) = 0.80 *P* = 0.45	–0.35
	TAU	1.59 (0.87)	1.50 (0.95)	1.75 (1.11)			
SCSQ MC	SCIT	9.04 (0.91)	9.41 (0.61)	9.35 (0.71)	*F*(2,123) = 2.14 *P* = 0.12	*F*(2,123) = 1.31 *P* = 0.27	0.33
	TAU	9.35 (0.70)	9.39 (0.64)	9.40 (0.61)			
Hinting Task	SCIT	12.69 (3.41)	14.09 (4.28)	14.88 (3.68)	*F*(2,124) = 401.13 *P* < 0.0001	*F*(2,124) = 1.76 *P* = 0.18	0.23
	TAU	14.28 (4.58)	15.16 (3.66)	15.53 (3.89)			
FEST	SCIT	66.01 (12.26)	73.15 (15.26)	68.73 (16.67)	*F*(2,124) = 6.07 *P* = 0.0031	*F*(2,124) = 1.41 *P* = 0.25	–0.05
	TAU	64.35 (13.50)	67.51 (12.97)	67.69 (15.33)			
BACS	SCIT	–1.64 (1.01)	–1.37 (1.09)	–1.13 (1.22)	*F*(2,124) = 25.31 *P* < 0.0001	*F*(2,124) = 0.57 *P* = 0.57	–0.05
	TAU	–1.33 (1.42)	–1.16 (1.36)	–0.77 (1.35)			
PANSS total	SCIT	64.78 (18.18)	61.34 (19.15)	60.03 (18.68)	*F*(2,124) = 5.86 *P* = 0.0037	*F*(2,124) = 0.10 *P* = 0.91	–0.04
	TAU	63.78 (18.98)	59.94 (18.02)	59.81 (17.32)			
PANSS P	SCIT	15.38 (5.13)	14.59 (5.58)	14.28 (4.79)	*F*(2,124) = 6.99 *P* = 0.0013	*F*(2,124) = 0.71 *P* = 0.49	0.18
	TAU	15.41 (5.87)	13.78 (5.19)	13.34 (5.00)			
PANSS N	SCIT	16.22 (5.38)	15.56 (5.09)	14.75 (5.27)	*F*(2,124) = 3.06 *P* = 0.05	*F*(2,124) = 0.55 *P* = 0.58	–0.10
	TAU	16.72 (5.86)	15.53 (6.21)	15.78 (5.36)			
PANSS G	SCIT	33.19 (9.82)	31.19 (10.07)	31.00 (10.18)	*F*(2,124) = 2.87 *P* = 0.06	*F*(2,124) = 0.37 *P* = 0.69	–0.13
	TAU	31.66 (9.70)	30.63 (9.03)	30.69 (8.83)			
GAF	SCIT	51.59 (8.81)	52.25 (13.63)	56.09 (15.33)	*F*(2,124) = 2.71 *P* = 0.07	*F*(2,124) = 2.23 *P* = 0.11	0.26
	TAU	53.44 (9.49)	58.06 (13.47)	55.59 (14.50)			
SFS	SCIT	116.06 (23.95)	118.44 (25.31)	117.45 (25.42)	*F*(2,121) = 0.63 *P* = 0.53	*F*(2,121) = 0.30 *P* = 0.74	–0.04
	TAU	106.53 (23.47)	107.41 (28.03)	108.91 (26.60)			
IMI	SCIT	N/A	104.68 (21.57)	100.10 (24.28)	*F*(1,57) = 0.63 *P* = 0.53	*F*(1,57) = 0.30 *P* = 0.74	–0.39
	TAU	N/A	89.30 (23.13)	93.97 (21.55)			

SCSQ, Social Cognition Screening Questionnaire; ToM, theory of mind; HB, hostility bias; MC, metacognition; FEST, Face Emotion Selection Test; BACS, Brief Assessment of Cognition in Schizophrenia; PANSS, Positive and Negative Syndrome Scale; GAF, Global Assessment of Functioning; SFS, Social Functioning Scale; IMI, Intrinsic Motivation Inventory; BL, baseline; 3M, 3-month timepoint; 6M, 6-month timepoint; SCIT, Social Cognition and Interaction Training; TAU, treatment as usual.

### Change in Other Measures of Social Cognition, Symptom Severity, Neurocognition, and Social Functioning

The results of the mixed effects modeling revealed no significant interaction between timepoint and group in any of the outcome measures, including social cognition, symptom severity, neurocognition, and social functioning (**Table 4**).

### Possible Predictors of Efficacy of SCIT for Social Cognition

Stepwise multiple regression analysis of the improvement in SCSQ total scores over the 6 months of treatment with SCIT, with sex, age, duration of illness, BACS score (baseline), SCSQ score (baseline), and IMI score (mean) as independent variables revealed that a low SCSQ (baseline) score and a short duration of illness significantly contributed to improvement in SCSQ total score (**Table 5**). Therefore, the full sample was subdivided according to the median value of the variables identified as predictors (median baseline SCSQ score: 32; median duration of illness: 149 months), and participants with total baseline SCSQ scores lower than or equal to 32 and those whose illness had a duration shorter than or equal to 149 months were submitted to secondary analyses using linear mixed effects modeling. Although there was no significant interaction between timepoint and group in the subgroup with low baseline SCSQ scores, a significant interaction was observed in the subgroup with a short duration of illness (**Table 6**). Specifically, this indicated that the change in total SCSQ scores between the baseline assessment and both the 3- and 6-month assessments was larger in the SCIT subgroup than in the TAU subgroup among participants with a relatively short duration of illness.

**Table 5:** Summary of stepwise multiple regression analysis on improvement in SCSQ total scores during 6 months of treatment with SCIT (dependent variable: change in SCSQ score over this period). Sex, age, duration of illness, BACS (baseline), SCSQ (baseline), and IMI (mean) were entered into the analysis as independent variables.

Variable	Standardized beta	*R* ^2^	*P*
SCSQ (baseline)	–0.52	0.34	0.001
Duration of illness	–0.01	0.16	0.011

SCSQ, Social Cognition Screening Questionnaire; SCIT, Social Cognition and Interaction Training; TAU, treatment as usual; BACS, Brief Assessment of Cognition in Schizophrenia; IMI, Intrinsic Motivation Inventory.

**Table 6:** Exploratory analysis in subgroups with low total SCSQ scores at baseline (≤32) and a short duration of illness (≤149 months). Both values represent the median in the full sample.

Median split subgroup	Subgroup	BL	3M	6M	Main effect of time	Time × group interaction	Effect size (6M–BL)
SCSQ total score ≤ 32 (*n* = 32)	SCIT (*n* = 19)	28.60 (2.29)	31.86 (3.10)	32.19 (3.35)	*F*(2,60) = 21.32 *P* < 0.0001	*F*(2,60) = 1.16 *P* = 0.32	0.57
	TAU (*n* = 13)	29.79 (3.24)	32.56 (2.56)	31.85 (4.43)			
Duration of illness ≤ 149 months (*n* = 32)	SCIT (*n* = 15)	31.20 (3.45)	33.56 (3.16)	34.04 (2.72)	*F*(2,59) = 8.31 *P* = 0.0007	*F*(2,59) = 3.17 *P* = 0.049	0.66
	TAU (*n* = 17)	33.02 (2.99)	33.53 (2.59)	33.71 (2.94)			

SCSQ, Social Cognition Screening Questionnaire; SCIT, Social Cognition and Interaction Training; TAU, treatment as usual; BL, baseline; 3M, 3-month timepoint; 6M, 6-month timepoint.

## Discussion

In this study, we translated, adapted, and evaluated SCIT for use with Japanese-speaking outpatients with schizophrenia. The persistence rate in the SCIT subgroup was relatively high, as only 4 out of 36 participants dropped out over the course of the whole program (persistence: 88.9%), and the average attendance rate for SCIT between the baseline and endpoint assessments was also high (87.0%, *SD* = 14.8). Both the persistence rate and the attendance rate in the present study were higher than those observed in previous community-based studies of SCIT (15, 41, 42). The relatively high IMI score in the SCIT subgroup compared with the TAU subgroup, especially in the first half of the program, indicates that the patients were well motivated. In fact, the IMI score observed in the SCIT subgroup (first half; 104.7 ± 21.6, second half; 100.1 ± 24.3) was much higher than that in Choi *et al*. (29), which presented a score of 61.14 ± 16.83 in the schizophrenia remediation sample. All in all, we found that SCIT appears to be feasible and well tolerated by patients with schizophrenia in Japan, as it has been when used with patients from other countries.

Regarding the social cognitive outcome measure (total SCSQ score), a significant change was observed between the baseline and 3-month interim assessments, and also between the baseline and 6-month endpoint assessments, only in the SCIT subgroup; however, the interaction between timepoint and group failed to reach significance, which suggests that the effect of SCIT was no different from that of TAU. These effectiveness results are not conclusive since the sample size may be too small, based on the meta-analytic study by Kurtz and Richardson (43). In their review of social cognitive interventions for schizophrenia, effect sizes varied from 0.07 to 1.01 across various subdomains of social cognition with a mean level of 0.40. If we used the effect size of 0.4, alpha set at 0.05, and power set at 0.8, the required sample size would be calculated to be 200. Again, as a first study in Japan, we decided the present study should be focused on feasibility and to generate effect size estimates for a future, more definitive study.

There was also no significant interaction between timepoint and group in their associations with any other measures of social cognition. Interestingly, most variables showed improvement between the baseline and 3-month interim timepoint, while changes between the 3-month interim timepoint and 6-month endpoint were small or absent. One possible explanation for this may be that a practice effect was more strongly in operation between the first two timepoints (baseline to 3-month interim timepoint) than between the second two timepoints (3-month interim timepoint to 6-month endpoint). A similar trend was observed in the TAU subgroup, which supports this view. Another possible explanation is that the treatment sessions in the latter half of the program were not as effective as expected, possibly because the tasks were too complex for some patients. In participants’ subjective reports, they rather often complained of difficulty in generating alternative views and differentiating between inferences and facts, activities that are included in the sessions that form the second half of the program. The decrease in IMI scores in the SCIT subgroup in the latter half of the program compared to the former may support this view at least in part. In addition, another possibility is that there was a ceiling effect on the SCSQ score. In fact, the mean level of the baseline SCSQ total score was 31.9 (SD = 3.6), which was slightly higher than the mean score obtained in our previous psychometric study (31.2, SD = 3.47) (31), and the distribution of the data was significantly skewed to the left (Shapiro-Wilk test, W = 0.96, P = 0.024).

The lack of effect on emotion perception, as measured by the FEST task, is inconsistent with previous research on SCIT (16, 17). Like the other measures of social cognition, participants’ FEST scores increased steeply at the 3-month interim assessment, but decreased substantially thereafter. Perceiving and understanding emotions are the primary focus of the first half of the sessions, and although the skill is revisited throughout the program in the form of “checking in,” the treatment is less intensive on this topic in the second half than in the first half. Thus, it may be beneficial to increase emotion perception training throughout the second half of the SCIT intervention program. In response to feedback and outcome data from previous studies, the developers of SCIT have revised several points in the treatment protocol manual (44). They have added partner exercises for practice and have also revised the SCIT content to make it easier for clients to digest and more flexible for clinicians to implement across a wide range of clinical settings in the current manual. These revisions were not present in the earlier version of the manual (28), which we followed in the present study. Further studies incorporating these new techniques are warranted in the future.

There was a significant interaction between timepoint and group when only patients with a relatively recent onset of illness were entered into the analysis, suggesting that SCIT may be more effective in patients with a shorter duration of illness. This finding needs to be interpreted with caution, since Kurtz and Richardson reported in a 2012 meta-analysis of interventions for social cognition that a longer duration of illness is associated with greater improvements in emotion perception and ToM (44). In the meta-analysis, participant samples were heterogeneous and included both inpatients and outpatients, which may explain this difference from the findings of the present study, in which only outpatients were recruited. A more recent systematic review suggests that studies highlighting the role of possible moderating factors, including areas such as illness chronicity, initial levels of functional or cognitive impairment, diagnosis, gender, and inpatient/outpatient status, would be important in developing a new generation of social cognition treatment programs (45). As some studies suggest that SCIT shows efficacy in patients with schizotypal personality and first-episode psychosis, early intervention for social cognition may well be effective in securing better outcomes in the future (19, 20). The period after recovery from a first episode of schizophrenia, extending over up to the subsequent 5 years, is known as the early course. If patients experience deterioration in symptoms and/or function, it is most likely to occur during this time, because by 5–10 years after onset, most patients experience a plateau in their level of illness and function, which is a so-called critical period (46, 47). It would be worthwhile to attempt to replicate our exploratory finding that patients with an onset of illness within the last 12 years, which overlaps with the critical period, may benefit from SCIT, because if this is the case, the program may well have the capacity to prevent functional deterioration.

The current study has a number of limitations. As noted above, the small sample size may have led to insufficient power to detect differences between the groups on outcome measures. Moreover, although as the first study to test the feasibility of SCIT in Japan we attempted to recruit the patients from a wide population, the patients who consented to participate in the study were relatively high functioning despite the moderate level of PANSS scores. Therefore, we could not conclude whether the program could be applied to lower functioning patients. Second, we did not account for changes to pharmacotherapy during the treatment period of this study. However, the potential effects of changes in pharmacotherapy on the efficacy assessment cannot be denied. Third, subjects’ participation in psychosocial treatments other than SCIT was not controlled, which may have caused confounding effects. Finally, the therapeutic durability of training effects was not assessed. All these limitations should be taken into consideration in interpreting the efficacy analyses; however, none impacts conclusions regarding the feasibility and acceptability of SCIT.

In summary, although this study showed limited benefits for social cognition outcome measures associated with SCIT compared with TAU, SCIT is feasible for use with and well tolerated by Japanese patients with schizophrenia in real-world outpatient settings.

## Ethics Statement

Ethical approval was obtained for the study (Ethical Committee of the National Centre of Neurology and Psychiatry) and the trial was registered with the University Hospital Medical Information Network (UMIN000011240; URL: https://upload.umin.ac.jp/cgi-open-bin/ctr/ctr_view.cgi?recptno=R000013171)

## Author Contributions

AKa, KI, EI, TN, KN, and DR were involved in the conceptualization and planning of the study. AKi, DH, YT, YY, AI, YM, TM, TF, ST, KH, and SO were involved in the implementation of the trial. AKa, AKi, KH, SO, and KN developed the Japanese version of the treatment intervention. AKa was responsible for management of the trial, including implementation of randomization procedures, data management, and drafting the manuscript. KN was responsible for the statistical analyses. All authors critically reviewed the manuscript and approved the final version.

## Funding

This study was supported by a National Center of Neurology and Psychiatry Intramural Research Grant (24–1, 27–1) for Neurological and Psychiatric Disorders.

## Conflict of Interest Statement

The authors declare that this research was conducted in the absence of any commercial or financial relationships that could be construed as a potential conflict of interest.
